# Prevalence and factors associated with antenatal depressive symptoms across trimesters: a study of 110,584 pregnant women covered by a mobile app-based screening programme in Shenzhen, China

**DOI:** 10.1186/s12884-024-06680-z

**Published:** 2024-07-16

**Authors:** Dadong Wu, Siqi Chen, Xiaoqi Zhong, Jiayi Zhang, Guanglin Zhao, Lei Jiang

**Affiliations:** 1 Shenzhen Maternity and Child Healthcare Hospital, Shenzhen, 518000 Guangdong Province China; 2 Shenzhen Key Laboratory of Maternal and Child Health and Diseases, Shenzhen, 518000 Guangdong Province China; 3 The Second School of Clinical Medicine, Zhujiang Hospital, Southern Medical University, Guangzhou, 510280 Guangdong Province China; 4 School of Health Management, Southern Medical University, Guangzhou, 510515 Guangdong Province China

**Keywords:** Antenatal depression, Prevalence, Associated factor, Pregnant women, Perinatal care, China

## Abstract

**Background:**

Antenatal depression is a significant public health issue affecting pregnant women both globally and in China. Using data from a mobile app-based screening programme, this study explored the prevalence and factors associated with antenatal depressive symptoms across different trimesters in Shenzhen.

**Methods:**

A retrospective cross-sectional study was conducted on pregnant women who gave birth in any hospital in Shenzhen between July 2021 and May 2022 and underwent depression screening using an official maternal and infant health mobile app at least once during pregnancy. Depressive symptoms were evaluated using the 9-item Patient Health Questionnaire (PHQ-9), with cut-off scores of 5 and 10 for mild and high level of symptoms, respectively. The prevalence for each trimester was determined by calculating the proportion of women scoring 5 or higher. A variety of sociodemographic, obstetric, psychological, and lifestyle factors were assessed for their association with depressive symptoms. Chi-square test and multivariate logistic regression were performed to identify significant predictors.

**Results:**

A total of 110,584 pregnant women were included in the study, with an overall prevalence of depressive symptoms of 18.0% and a prevalence of high-level symptoms of 4.2%. Depressive symptoms were most prevalent in the first trimester (10.9%) and decreased in the second (6.2%) and third trimesters (6.3%). Only a small proportion (0.4%) of women showed persistent depressive symptoms across all trimesters. Anxiety symptoms in early pregnancy emerged as the most significant predictor of depressive symptoms. Other factors linked to an increased risk throughout pregnancy include lower marital satisfaction, living with parents-in-law, experience of negative life events, as well as drinking before and during pregnancy. Factors associated with a reduced risk throughout pregnancy include multiparity and daily physical activity.

**Conclusions:**

This large-scale study provides valuable insights into the prevalence and factors associated with antenatal depressive symptoms in Shenzhen. The findings underscore the need for targeted interventions for high-risk groups and the integration of mental health care into routine antenatal services. Continuous, dynamic monitoring of depressive symptoms for pregnant women and ensuring at-risk women receive comprehensive follow-up and appropriate psychological or psychiatric care are crucial for effectively addressing antenatal depression and improving maternal and infant health outcomes.

## Background

Antenatal depression, the most prevalent mental disorder in pregnancy, presents a major public health challenge [1]. According to a recent systematic review of systematic reviews, approximately 28.5% of pregnant women worldwide are affected by depressive symptoms to varying extents [2]. These conditions not only adversely affect the quality of life of pregnant women but, if left untreated, also have serious implications for maternal and infant health. Antenatal depression is associated with a range of negative health consequences, including preeclampsia, operative deliveries (such as caesarean sections and instrumental vaginal deliveries), preterm birth, low birth weight, postpartum depression, and an increased risk of maternal suicide [3–7]. Compared to pregnant women without depressive symptoms, those with high level of depressive symptoms face a 2.26 times higher overall risk of adverse birth outcomes, such as preterm birth and low birth weight [8]. Furthermore, maternal depressive symptoms can negatively impact the behavioural and cognitive development of offspring, thereby placing a substantial long-term burden on the family and society [9].

Despite its high prevalence and significant consequences, antenatal depression has received less attention than postpartum depression [10]. This lack of focus leads to frequent underdiagnosis and undertreatment [11], amplifying its status as a key public health problem. In recent years, major medical associations, such as the American College of Obstetricians and Gynecologists (ACOG), have strongly advocated for routine depression screening during pregnancy [12]. This strategy, combined with the diagnosis and management of those who tested positive, is recognised as an effective way to mitigate the burden of antenatal depression while addressing barriers faced by patients, clinicians, and healthcare systems [13]. In September 2020, the National Health Commission of China introduced the first national guideline for the prevention and treatment of perinatal depression, which recommend the integration of depression screening into routine antenatal care, specifically utilising the 9-item Patient Health Questionnaire (PHQ-9) [14]. This recommendation was based on the Chinese version of the PHQ-9 being a valid and efficient tool that has been extensively tested in various Chinese populations, including pregnant women [15, 16]. However, evidence regarding the adoption of the national guideline at local levels remains limited.

Numerous studies have investigated the prevalence and associated factors of antenatal depressive symptoms among Chinese women. A systematic review by Nisar et al. reported the pooled prevalence in Mainland China to be 19.7%, with trimester-specific prevalences of 7.4%, 12.8%, and 12.0% for the first, second, and third trimester, respectively [17]. While these rates are lower than those reported in many other low- and middle-income countries (ranging from 15 to 65%) [8], the burden of antenatal depressive symptoms in China remains significant due to its large population. The factors associated with depressive symptoms during pregnancy, as identified in existing literature, typically fall into sociodemographic, obstetric, and psychological categories. These include the pregnant woman’s age, education level, place of residence, employment status, marital satisfaction, family economic situation, anxiety symptoms during pregnancy, perceived stress, negative life events, and relationships with parents and parents-in-law [18–21]. However, there is a lack of broad, population-based research on the prevalence of antenatal depressive symptoms and their associated factors across different trimesters. Furthermore, compared to international studies [22–24], the exploration into how lifestyle factors like smoking, alcohol use, and sleep quality associate with antenatal depressive symptoms in China has not been extensive. These gaps in research highlight the limitations in current understanding and underscore the need for more comprehensive studies to inform the effective planning of routine screening for antenatal depression.

In 2021, Shenzhen, a highly developed city in Southern China with a population of about 17.5 million, initiated a pioneering programme that provides mobile app-based depression screening for all pregnant women during antenatal care. During their antenatal visits, pregnant women are guided by healthcare professionals to complete the PHQ-9 screening using an official maternal and infant health mobile app (“Fu-Er-Tong”) in their first, second, and third trimester, respectively. As required by the programme guideline [25], those who screen positive (PHQ-9 score ≥ 5) are then referred to clinical psychology departments for psychosocial and psychological interventions. Those with severe depressive symptoms, such as suicidal ideation, are referred to the municipal psychiatric specialty hospital or psychiatric departments within general hospitals for treatment. The data collected from this programme is automatically input into and managed by a specific module within the municipal Maternal and Child Health Management Information System (MCHMIS). The utilisation of mobile technology and the health information system has not only facilitated the expansion of screening coverage but also the generation of higher-quality data [26]. Utilising the MCHMIS data, the present study was conducted to explore the prevalence and factors associated with antenatal depressive symptoms in Shenzhen. It aims to provide population-based evidence that contributes to expanding the knowledge base of antenatal depression, thereby supporting informed public health strategies.

## Methods

### Study population

A retrospective cross-sectional study was conducted on pregnant women who gave birth in any of the 82 midwifery hospitals across 10 districts in Shenzhen between July 2021 and May 2022 and had depression screening results available for at least one of the three trimesters. This time frame was chosen because the women initiated their first antenatal visits from January 2021, coinciding with the launch of “Fu-Er-Tong”. All women, regardless of their residence status, were covered by basic maternal and infant health services, making their individual case data accessible from the MCHMIS. Women who did not give birth in Shenzhen were excluded due to the inability to accurately track their follow-up data and birth outcomes. For this study, a total of 113,652 electronic records were exported from the MCHMIS. After a thorough review and correction of any logistic errors, 110,584 (97.3%) were included for statistical analysis. This sample represents 82.3% of all women who gave birth in Shenzhen during this period (*n* = 134,308). Notably, this rate is close to the perinatal depression screening coverage in Shenzhen prior to the COVID-19 pandemic [27], suggesting that the pregnant women in this study were not likely affected by the pandemic in terms of their participation in the screening programme. This could be due to the routine screening approach and effective use of mobile technology, which may have broadened participation and altered self-reporting behaviours during the social restrictions [28].

### Data collection and management

Data collection was primarily conducted by healthcare professionals in obstetrical departments across all the 10 districts in Shenzhen. They assisted pregnant women in self-rating their depressive symptoms using an electronic PHQ-9 available in the mobile app during their antenatal visits. According to the programme guideline, each woman was screened three times: once in each of the three trimesters. In addition, a pre-designed electronic questionnaire was used to gather comprehensive information on each woman's sociodemographic background, obstetric history, psychological factors, and lifestyle choices. This data, along with the women's PHQ-9 scores, were then automatically integrated into their maternal and infant health records within the MCHMIS. Shenzhen Maternity and Child Healthcare Hospital was responsible for coordinating the screening programme. To enhance programme implementation and data quality, the hospital organised periodic trainings and monitoring activities for relevant healthcare professionals. For this study, the original data was exported from the MCHMIS and managed by authorised personnel, ensuring confidentiality. Access to the study database was restricted to the authors for the purpose of this study, adhering to strict data privacy and security protocols.

### Assessment of associated factors

The questionnaire collected data on various sociodemographic, obstetric, psychological, and lifestyle variables of the pregnant women. These factors, potentially associated with antenatal depressive symptoms, were identified through literature review [18–24] and expert consultation, and were mostly assessed using single questions. Sociodemographic variables included age, employment status (white collar worker, blue collar worker, self-employed, housewife, others), education level (junior school and below, high school, bachelor/college, master and above), annual family income in Chinse yuan (CNY) (below 120,000, 120,000 ~ 240,000, above 240,000), and living condition (nuclear family, living with parents, living with parents-in-law, and living alone). Obstetric variables covered gravidity, parity, pregnancy planning status (unplanned, planned), and conception type (natural, assisted). Psychological variables encompassed marital satisfaction (satisfied, neither satisfied nor unsatisfied, unsatisfied), anxiety symptoms in the first trimester (assessed by the 7-item General Anxiety Disorder scale (GAD-7) with a cut-off score of 5 indicating mild or more severe anxiety symptoms, which has been proven reliable and valid for Chinese pregnant women, especially in early pregnancy [16, 29]), family history of mental illness, and experience of negative life events (e.g., divorce, job loss, serious illness or death of family members etc.) during pregnancy. Lifestyle variables asked about tobacco and alcohol use habits of the women and their husbands. Additionally, physical activity during pregnancy was assessed with responses categorised as “never”, “occasionally”, “weekly”, and “daily”.

### Measure of depressive symptoms

The women’s depressive symptoms were measured using the validated Chinese version of PHQ-9 [15]. Cut-off scores of 5 and 10 were utilised to indicate mild and high level of depressive symptoms, respectively. These thresholds are internationally recognised and have been validated among Chinese pregnant women [16, 30]. Both the PHQ-9 and GAD-7 have been validated for use in various formats, including mobile app and remote administration. Studies have shown that these tools retain their reliability and validity when administered electronically [31, 32].

### Statistical analysis

The statistical analysis was performed using R version 4.0.2. Initially, the prevalence of antenatal depressive symptoms for each trimester was assessed by calculating the proportion of pregnant women screened positive (PHQ-9 score ≥ 5). Chi-square tests were then applied to determine the differences in prevalence across trimesters. Subsequently, frequencies of each potential associated factor in different trimesters were calculated. For univariate analysis, Chi-square tests were utilised to compare these factors between women with positive depression screening results and those without. A *p*-value of less than 0.05 was considered to indicate statistical significance. For all variables that showed significant differences, a multivariate logistic regression analysis was conducted to estimate Odds ratios (OR) and 95% Confidence intervals (95% CI), with a *p*-value for statistical significance set at 0.05. In the logistic regression model, the first level of each ordered categorical variable was used as the reference category. Unordered categorical variables, such as employment status and living condition, were converted into dummy variables. Within these, white collar workers and those living in nuclear family were selected as the reference groups. We used complete case analysis, excluding any records with missing values from the analysis, to ensure the accuracy and integrity of the results.

## Results

### Prevalence of depressive symptoms across trimesters

Among the 110,584 women studied, a total of 19,852 were screened positive (PHQ-9 score ≥ 5) at least once during the three trimesters, demonstrating an overall prevalence of 18.0%. Additionally, 4619 (4.2%) women recorded PHQ-9 scores of 10 or higher at least once, which points to a high level of depressive symptoms. Of note, only 450 (0.4%) women were screened positive across all trimesters. In terms of screening completion, 80,626 (60.0%) women completed the screening in the first trimester, 76,711 (57.1%) in the second trimester, and 78,524 (58.5%) in the third trimester. The difference in screening completion rates across trimesters is statistically significant (*χ*^*2*^ = 2489.28, *p* < 0.001). As shown in Table 1, the prevalence of mild depressive symptoms was 10.9% in the first trimester, 6.2% in the second trimester, and slightly rising to 6.3% in the third trimester. The prevalence of high-level depressive symptoms was 3.4% in the first trimester, which decreased to 1.5% in the second trimester and further to 1.4% in the third trimester. Chi-square test revealed statistically significant differences in pregnant women’s screening results across trimester (*χ*^*2*^ = 2664.73, *p* < 0.001).

**Table 1:** Depression screening results across trimesters of the 110,584 pregnant women

PHQ-9	First trimester	Second trimester	Third trimester	*χ*^*2*^	*p*
Screening completion	80,626 (60.0%)	76,711 (57.1%)	78,524 (58.5%)	2489.28	< 0.001
Score < 5	69,118(85.7%)	70,798 (92.3%)	72,476 (92.3%)	2664.73	< 0.001
5 ≤ score < 10	8757 (10.9%)	4795(6.2%)	4940 (6.3%)		
Score ≥ 10	2751 (3.4%)	1118 (1.5%)	1108 (1.4%)		

*PHQ-9* 9-item Patient Health Questionnaire

### Sociodemographic factors associated with antenatal depressive symptoms

The average age of the 110,584 pregnant women at their first screening was 29.8 (standard deviation: 4.4, range: 16 ~ 51). About three-fourths (73.3% ~ 73.9%) aged between 26 and 35. More than half (55.7% ~ 56.7%) were employees of government/public enterprises or white-collar workers, 15.2% ~ 15.7% were business owners or self-employed, 14.6% ~ 15.4% were housewives, and 7.4% ~ 8.0% were blue-collar workers, soldiers, or farmers. Nearly two-thirds (60.1% ~ 61.4%) of the women had attained bachelor’s degree or higher. About half (50.8 ~ 53.8) reported an annual family income exceeding CNY 240,000. Most women (76.2% ~ 78.0%) resided in nuclear families, 16.4% ~ 17.9 lived with parents-in-law, 3.8% ~ 4.3% lived with their own parents, and 1.6% ~ 1.7% lived alone.

As depicted in Table 2, depressive symptoms were more prevalent among pregnant women aged 26 ~ 35, those with bachelor’s degree or higher, those with an annual family income below CNY 240,000, and those employed by government/public enterprises or as white-collar workers across all trimesters. Women living with parents or parents-in-law reported higher PHQ-9 scores compared to those living in nuclear families or alone. The univariate analysis revealed statistically significant differences in most of the aforementioned factors.

**Table 2:** Sociodemographic factors associated with antenatal depressive symptoms across trimesters among the 110,584 pregnant women

Sociodemographic variables	First trimester (week 1 ~ 13, *N* = 80,626)				Second trimester (week 14–27, *N* = 76,711)				Third trimester (week 28–40, *N* = 78,524)			
	**n (%)**	**AD (%)**	***χ***^**2**^	**OR [95% CI]**	**n (%)**	**AD (%)**	***χ***^**2**^	**OR [95% CI]**	**n (%)**	**AD (%)**	***χ***^**2**^	**OR [95% CI]**
**Age group**			71.08*				15.86*				55.85*	
26 ~ 35	59,563 (73.9)	8843 (76.8)		1.00 [ref]	56,225 (73.3)	4341 (73.4)		1.00 [ref]	57,843 (73.7)	4498 (74.4)		1.00 [ref]
16 ~ 25	12,821 (15.9)	1700 (14.8)		0.80 [0.72 ~ 0.88]*	12,844 (16.7)	1059 (17.9)		0.91 [0.78 ~ 1.05]	13,089 (16.7)	1117 (18.5)		0.92 [0.81 ~ 1.05]
≥ 36	8242 (10.2)	965 (8.4)		0.99 [0.87 ~ 1.13]	7642 (10.0)	513 (8.7)		1.17 [0.96 ~ 1.42]	7592 (9.7)	433 (7.2)		0.91 [0.75 ~ 1.10]
**Employment status**			489.63*				179.69*				120.46*	
Employee of government/public enterprises or white-collar worker	45,685 (56.7)	7211 (62.7)		1.00 [ref]	42,743 (55.7)	3418 (57.8)		1.00 [ref]	43,817 (55.8)	3479 (57.5)		1.00 [ref]
Business/Self-employed	12,249 (15.2)	1196 (10.4)		0.83 [0.74 ~ 0.92]*	12,080 (15.7)	890 (15.1)		1.48 [1.29 ~ 1.70]*	119,51 (15.2)	916 (15.1)		1.34 [1.17 ~ 1.52]*
Blue-collar worker/ soldier/farmer	5994 (7.4)	639 (5.6)		0.95 [0.83 ~ 1.09]	5932 (7.7)	283 (4.8)		0.70 [0.55 ~ 0.88]*	6245 (8.0)	331 (5.5)		0.71 [0.58 ~ 0.87]*
Housewife	11,740 (14.6)	1492 (13.0)		1.11 [0.99 ~ 1.23]	11,823 (15.4)	837 (14.2)		0.99 [0.83 ~ 1.18]	12,050 (15.3)	843 (13.9)		1.01 [0.87 ~ 1.17]
Others	4958 (6.1)	970 (8.4)		1.56 [1.36 ~ 1.79]*	4133 (5.4)	485 (8.2)		1.11 [0.85 ~ 1.42]	4461 (5.7)	479 (7.9)		1.33 [1.08 ~ 1.62]*
**Education level**			400.23*				88.88*				37.51*	
Secondary school and below	13,200 (16.4)	1263 (11.0)		1.00 [ref]	13,584 (17.7)	808 (13.7)		1.00 [ref]	13,630 (17.4)	891 (14.7)		1.00 [ref]
High school	13,774 (17.1)	1712 (14.9)		1.15 [1.01 ~ 1.31]*	13,432 (17.5)	975 (16.5)		1.15 [0.94 ~ 1.40]	13,516 (17.2)	1007 (16.7)		1.14 [0.97 ~ 1.35]
Bachelor/college	49,515 (61.4)	7820 (68.0)		1.60 [1.43 ~ 1.79]*	46,116 (60.1)	3844 (65.0)		1.38 [1.16 ~ 1.64]*	47,465 (60.4)	3839 (63.5)		1.12 [0.97 ~ 1.31]
Master and above	4137 (5.1)	713 (6.2)		2.07 [1.70 ~ 2.51]*	3579 (4.7)	286 (4.8)		1.71 [1.26 ~ 2.30]*	3913 (5.0)	311 (5.1)		1.10 [0.82 ~ 1.45]
**Living condition**			472.94*				434.91*				291.46*	
Nuclear family	38,687 (77.7)	4228 (67.2)		1.00 [ref]	38,126 (78.0)	2585 (65.0)		1.00 [ref]	38,635 (76.2)	2671 (65.4)		1.00 [ref]
Living with parents	1916 (3.8)	357 (5.7)		1.31 [1.16 ~ 1.53]*	1946 (4.0)	223 (5.6)		1.11 [0.86 ~ 1.41]	2176 (4.3)	243 (5.9)		1.34 [1.08 ~ 1.64]*
Living with in-laws	8380 (16.8)	1531 (24.3)		1.49 [1.36 ~ 1.62]*	8011 (16.4)	1041 (26.2)		1.72 [1.51 ~ 1.95]*	9064 (17.9)	1053 (25.8)		1.46 [1.30 ~ 1.64]**
Living alone	792 (1.6)	177 (2.8)		1.34 [1.05 ~ 1.69]*	796 (1.6)	124 (3.1)		1.12 [0.77 ~ 1.59]	855 (1.7)	119 (2.9)		1.13 [0.80 ~ 1.54]
**Annual family income**			839.39*				710.91*				584.75*	
Below CNY 120,000	272 (0.5)	83 (1.3)		1.00 [ref]	313(0.6)	72 (1.8)		1.00 [ref]	358 (0.7)	93 (2.3)		1.00 [ref]
CNY 120,000 ~ 240,000	22,696 (45.6)	3864 (61.4)		0.78 [0.53 ~ 1.17]	22,632 (46.3)	2564 (64.5)		1.12 [0.64 ~ 2.08]	24,584 (48.5)	2581 (63.2)		1.20 [0.67 ~ 1.98]
Above CNY 240,000	26,800 (53.8)	2347 (37.3)		0.52 [0.35 ~ 0.78]*	25,945 (53.1)	1337 (33.7)		0.74 [0.43 ~ 1.39]	25,794 (50.8)	1413 (34.6)		0.80 [0.48 ~ 1.41]

*N* Number, *AD* Antenatal depression, *OR* Odds ratio, *CI* Confidence interval

^*^p < 0.05, ^**^p < 0.001

### Psychological factors associated with antenatal depressive symptoms

A vast majority of the pregnant women (93.5% ~ 94.5%) expressed satisfaction with their marriage, 5.4% ~ 6.3% were neither satisfied nor unsatisfied, and a minimal proportion (0.2%) were unsatisfied. Only 1.0% ~ 1.1% reported experiencing negative life events during pregnancy, and 0.3% had a family history of mental illness. In addition, 6.2% were found to have some extent of anxiety symptoms in early pregnancy.

According to the Chi-square test results presented in Table 3, although relatively infrequent, the prevalence of depressive symptoms was significantly higher among pregnant women with existing psychological issues throughout the three trimesters. These issues include a family history of mental illness, anxiety symptoms in early pregnancy, and negative life events during pregnancy. Additionally, women who were satisfied with their marriage reported lower PHQ-9 scores compared to those with lower marital satisfaction.

**Table 3:** Psychological factors associated with antenatal depressive symptoms across trimesters among the 110,584 pregnant women

Psychological variables	First trimester (week 1–13, *N* = 80,626)				Second trimester (week 14–27, *N* = 76,711)				Third trimester (week 28–40, *N* = 78,524)			
	**n (%)**	**AD (%)**	***χ***^**2**^	**OR [95% CI]**	**n (%)**	**AD (%)**	***χ***^**2**^	**OR [95% CI]**	**n (%)**	**AD (%)**	***χ***^**2**^	**OR [95% CI]**
**Marital satisfaction**			374.87*				413.27*				468.85*	
Satisfied	46,858 (94.5)	5608 (89.2)		1.00 [ref]	45,764 (93.9)	3437 (86.6)		1.00 [ref]	47,267 (93.5)	3496 (85.6)		1.00 [ref]
Neither satisfied nor unsatisfied	2673 (5.4)	660 (10.5)		1.31 [1.14 ~ 1.50]*	2883 (5.9)	514 (13.0)		1.47 [1.22 ~ 1.78]*	3205 (6.3)	558 (13.7)		1.56 [1.31 ~ 1.84]*
Unsatisfied	76 (0.2)	16 (0.3)		0.45 [0.16 ~ 1.21]	78 (0.2)	19 (0.5)		1.66 [0.52 ~ 4.36]	91 (0.2)	28 (0.7)		2.57 [1.00 ~ 5.92]*
**Anxiety symptoms in early pregnancy**			18,348*				3304.2*				2156.9*	
No	68,284 (93.8)	6706 (64.2)		1.00 [ref]	50,080 (94.9)	1844 (70.9)		1.00 [ref]	50,886 (94.6)	2374 (76.4)		1.00 [ref]
Yes	4505 (6.2)	3733 (35.8)		66.46 [58.70 ~ 75.47]*	2664 (5.1)	758 (29.1)		6.21 [5.42 ~ 7.10]*	2882 (5.4)	733 (23.6)		4.92 [4.33 ~ 5.58]*
**Family history of mental illness**			**1.090**				5.839*				5.969*	
No	75,713 (99.7)	10,797 (99.6)			71,930 (99.7)	5531 (99.5)		1.00 [ref]	73,592 (99.7)	5687 (99.5)		1.00 [ref]
Yes	231 (0.3)	39 (0.4)			210 (0.3)	26 (0.5)		1.80 [0.84 ~ 3.51]	218 (0.3)	27 (0.5)		0.90[0.36 ~ 1.91]
**Negative life event**			89.546*				97.125*				110.56*	
No	75,115 (99.0)	10,628 (98.1)		1.00 [ref]	71,296 (98.9)	5424 (97.5)		1.00 [ref]	72,928 (98.9)	5564 (97.4)		1.00 [ref]
Yes	786 (1.0)	205 (1.9)		1.47 [1.07 ~ 2.02]*	815 (1.1)	138 (2.5)		2.19 [1.48 ~ 3.16]*	836 (1.1)	146 (2.6)		2.12 [1.47 ~ 2.99]*

*N* Number, *AD* Antenatal depression, *OR* Odds ratio, *CI* Confidence interval

^*^p < 0.05

### Obstetric factors associated with antenatal depressive symptoms

The maternal health records indicated that 67.1% ~ 67.5% of the women had experienced 1 or 2 pregnancies. Of these, 49.1% ~ 49.3% had no child and 40.0% ~ 40.4% had 1 child prior to the current pregnancy. About one-third (32.2% ~ 33.0%) of the women reported that the current pregnancy was unplanned, while 3.9% ~ 4.3% underwent assisted conception.

Table 4 shows that women with fewer pregnancies (gravidity) and fewer births (parity) tended to report higher PHQ-9 scores across all trimesters. Furthermore, depressive symptoms were more prevalent among those with unplanned pregnancies or who underwent assisted conception.

**Table 4:** Obstetric factors associated with antenatal depressive symptoms across trimesters among the 110,584 pregnant women

Obstetric variables	First trimester (week 1–13, *N* = 80,626)				Second trimester (week 14–27, *N* = 76,711)				Third trimester (week 28–40, *N* = 78,524)			
	**n (%)**	**AD (%)**	***χ***^**2**^	**OR [95% CI]**	**n (%)**	**AD (%)**	***χ***^**2**^	**OR [95% CI]**	**n (%)**	**AD (%)**	***χ***^**2**^	**OR [95% CI]**
**Gravidity**			200.87*				91.93*				91.05*	
1	27,491 (35.6)	4474 (41.4)		1.00 [ref]	25,900 (35.6)	2342 (41.4)		1.00 [ref]	26,610 (36.1)	2396 (41.6)		1.00 [ref]
2	24,481 (31.7)	3283 (30.4)		0.93 [0.83 ~ 1.04]	22,878 (31.5)	1675 (29.6)		0.96 [0.81 ~ 1.13]	23,128 (31.4)	1747 (30.3)		0.90 [0.77 ~ 1.04]
3	14,019 (18.2)	1719 (15.9)		1.03[0.89 ~ 1.17]	13,306 (18.3)	906 (16.0)		0.89 [0.72 ~ 1.09]	13,324 (18.1)	914 (15.9)		0.86 [0.71 ~ 1.04]
≥ 4	11,130 (14.4)	1325 (12.3)		1.09 [0.93 ~ 1.28]	10,569 (14.5)	734 (13.0)		1.00 [0.79 ~ 1.27]	10,637 (14.4)	708 (12.3)		0.92 [0.74 ~ 1.14]
**Parity**			532.01*				209.69*				122.27*	
0	37,929 (49.3)	6352 (58.8)		1.00 [ref]	35,579 (49.1)	3270 (57.8)		1.00 [ref]	36,241 (49.3)	1,240 (59.5)		1.00 [ref]
1	31,050 (40.4)	3757 (34.8)		0.73 [0.65 ~ 0.81]*	28,972 (40.0)	1964 (34.7)		0.82 [0.69 ~ 0.97]*	29,385 (40.0)	728 (34.9)		0.80 [0.69 ~ 0.94]*
2	6951 (9.0)	636 (5.9)		0.58 [0.48 ~ 0.70]*	6920 (9.5)	375 (6.6)		0.61 [0.45 ~ 0.81]*	6797 (9.3)	105 (5.0)		0.56 [0.43 ~ 0.73]*
≥ 3	984 (1.3)	49 (0.5)		0.19 [0.11 ~ 0.32]*	1039 (1.4)	45 (0.8)		0.46 [0.21 ~ 0.89]*	1054 (1.4)	12 (0.6)		0.48 [0.25 ~ 0.84]*
**Unplanned pregnancy**			56.41*				27.49*				70.88*	
No	33,846 (67.8)	4008 (63.7)		1.00 [ref]	32,828 (67.0)	2359 (59.4)		1.00 [ref]	34,055 (67.0)	2492 (61.0)		1.00 [ref]
Yes	16,073 (32.2)	2288 (36.3)		1.09 [1.00 ~ 1.17]*	16,186 (33.0)	1610 (40.6)		1.21 [1.08 ~ 1.36]*	16,794 (33.0)	1592 (39.0)		1.09 [0.98 ~ 1.21]
**Conception type**			60.38*				54.55*				12.42*	
Natural	48,124 (96.1)	5945 (94.3)		1.00 [ref]	47,101 (95.7)	3716 (93.4)		1.00 [ref]	48,856 (95.7)	3869 (94.8)		
Assisted	1979 (3.9)	362 (5.7)		1.16 [0.98 ~ 1.37]	2105 (4.3)	261 (6.5)		1.38 [1.09 ~ 1.73]*	2171 (4.3)	218 (5.3)		0.94 [0.74 ~ 1.19]

*N* Number, *AD* Antenatal depression, *OR* Odds ratio, *CI* Confidence interval

^*^p < 0.05

### Lifestyle factors associated with antenatal depressive symptoms

The majority of the women never smoked (97.9% ~ 98.1%) or drank (83.2% ~ 84.6%). These rates are consistent with the those reported in published studies [33, 34]. Only 1.0% ~ 1.2% smoked and 4.1% ~ 5.1% drank solely before pregnancy, while 0.8% ~ 0.9% continued to smoke and 11.2% ~ 11.7% continued to drink during pregnancy. Regarding physical activity, 47.0% ~ 49.6% of the women engaged in exercise occasionally, 16.3% ~ 18.2% weekly, 10.4% ~ 11.5% daily, while 21.9% ~ 24.2% did not exercise at all during pregnancy. Concerning their husbands’ habits, 60.8%-61.6% never smoked and 50.7%-51.6% never drank, 19.6%-19.9% smoked and 41.7%-42.3% drank only before their wives’ pregnancies, while 19.0%-20.2% continued to smoke and 6.7%-7.0% continue to drink during their wives’ pregnancies.

Throughout the three trimesters, as indicated in Table 5, women who smoked or drank, either before or during pregnancy, reported higher PHQ-9 scores compared to women who never smoked or drank. Similarly, husbands’ smoking and drinking habits were associated with higher PHQ-9 scores of the wives. Conversely, a higher frequency of exercise was associated with a lower likelihood of depressive symptoms. All these differences were statistically significant in the univariate analysis.

**Table 5:** Lifestyle factors associated with antenatal depressive symptoms across trimesters among the 110,584 pregnant women

Lifestyle variables	First trimester (week 1–13, *N* = 80,626)				Second trimester (week 14–27, *N* = 76,711)				Third trimester (week 28–40, *N* = 78,524)			
	**n (%)**	**AD (%)**	***χ***^**2**^	**OR [95% CI]**	**n (%)**	**AD (%)**	***χ***^**2**^	**OR [95% CI]**	**n (%)**	**OR [95% CI]**	***χ***^**2**^	**OR [95% CI]**
**Smoking**			213.68*				243.55*				151.61*	
Never	48,916 (98.1)	6029 (95.8)		1.00 [ref]	47,990 (98.0)	3766 (94.8)		1.00 [ref]	49,755 (97.9)	4134 (96.0)		1.00 [ref]
Before pregnancy	519 (1.0)	153 (2.4)		1.13 [0.85 ~ 1.47]	554 (1.1)	135 (3.3)		1.80 [1.28 ~ 2.50]*	614 (1.2)	99 (3.0)		1.17 [0.83 ~ 1.63]
During pregnancy	408 (0.8)	110 (1.7)		1.43 [1.04 ~ 1.93]*	414 (0.8)	75 (1.9)		1.69 [1.12 ~ 2.49]*	433 (0.9)	75 (1.7)		1.11 [0.72 ~ 1.66]
**Drinking**			1621.7*				1125.6*				848.23*	
Never	42,131 (84.6)	4242 (67.5)		1.00 [ref]	41,277 (84.4)	2620 (66.0)		1.00 [ref]	42,206 (83.2)	2736 (67.0)		1.00 [ref]
Before pregnancy	2052 (4.1)	560 (8.9)		1.96 [1.70 ~ 2.26]*	2184 (4.5)	428 (10.8)		1.83 [1.49 ~ 2.23]*	2605 (5.1)	458 (11.2)		1.87 [1.56 ~ 2.24]*
During pregnancy	5616 (11.3)	1485 (23.6)		1.82 [1.66 ~ 2.01]*	5464 (11.2)	924 (23.3)		1.60 [1.39 ~ 1.85]*	5945 (11.7)	892 (21.8)		1.44 [1.26 ~ 1.64]*
**Physical activity**			1303.1*				530.53*				127.48*	
Never	11,929 (24.2)	2589 (41.3)		1.00 [ref]	10,587 (21.9)	1385 (35.0)		1.00 [ref]	11,344 (22.6)	1192 (29.3)		1.00 [ref]
Occasionally	23,248 (47.2)	2672 (42.7)		0.59 [0.55 ~ 0.64]*	23,692 (48.9)	1864 (47.0)		0.84 [0.75 ~ 0.95]*	24,931 (49.6)	1907 (46.8)		0.93 [0.83 ~ 1.04]
Weekly	8970 (18.2)	611 (9.8)		0.38 [0.33 ~ 0.42]*	8809 (18.2)	478 (12.0)		0.60 [0.50 ~ 0.73]*	8209 (16.3)	617 (15.2)		0.91 [0.78 ~ 1.07]
Daily	5142 (10.4)	391 (6.2)		0.48 [0.42 ~ 0.55]*	5317 (11.0)	235 (5.9)		0.41 [0.31 ~ 0.52]*	5763 (11.5)	356 (8.7)		0.68 [0.56 ~ 0.83]*
**Husband smoking**			130.19*				211.47*				136.1*	
Never	30,644 (61.5)	3536 (56.2)		1.00 [ref]	29,745 (60.8)	2054 (51.7)		1.00 [ref]	31,297 (61.6)	2196 (53.7)		1.00 [ref]
Before pregnancy	9727 (19.5)	1243 (19.8)		0.87 [0.79 ~ 0.96]*	9586 (19.6)	808 (20.3)		0.90 [0.78 ~ 1.04]	9232 (18.2)	812 (19.9)		1.34 [1.19 ~ 1.51]*
During pregnancy	9461 (19.0)	1513 (24.0)		0.94 [0.85 ~ 1.03]	9623 (19.6)	1111 (28.0)		0.93 [0.80 ~ 1.1]	10,266 (20.2)	1079 (26.4)		1.39 [1.14 ~ 1.70]*
**Husband drinking**			851.89*				704.41*				540.49*	
Never	25,618 (51.6)	2163 (34.4)		1.00 [ref]	24,819 (50.9)	1220 (30.7)		1.00 [ref]	25,620 (50.7)	1359 (33.3)		1.00 [ref]
Before pregnancy	20,682 (41.7)	3548 (56.5)		1.49 [1.36 ~ 1.62]*	20,596 (42.2)	2344 (59.0)		1.57 [1.38 ~ 1.80]*	21,414 (42.3)	2307 (56.5)		0.95 [0.83 ~ 1.08]
During pregnancy	3325 (6.7)	572 (9.1)		1.47 [1.26 ~ 1.71]*	3359 (6.9)	406 (10.2)		1.61 [1.29 ~ 2.02]*	3541 (7.0)	415 (10.2)		1.02 [0.89 ~ 1.17]

*N* Number, *AD* Antenatal depression, *OR* Odds ratio, *CI* Confidence interval

^*^p < 0.05

### Multivariate logistic regression

All variables that demonstrated significant differences between the pregnant women with positive depression screening results and those without were included in the multivariate logistic regression model. Six factors were consistently identified as significantly associated with an increased risk of depressive symptoms across all trimesters. These include living with parents-in-law (First trimester: OR = 1.49, 95%CI 1.36 ~ 1.62; Second trimester: OR = 1.72, 95%CI: 1.51 ~ 1.95; Third trimester: OR = 1.46, 95%CI: 1.30 ~ 1.64), lower marital satisfaction (First trimester: OR = 1.31, 95%CI: 1.14 ~ 1.50; Second trimester: OR = 1.47, 95%CI: 1.22 ~ 1.78; Third trimester: OR = 1.56 95%CI: 1.31 ~ 1.84), anxiety symptoms in early pregnancy (First trimester: OR = 66.46, 95%CI: 58.70 ~ 75.47; Second trimester: OR = 6.21, 95%CI: 5.42 ~ 7.10; Third trimester: OR = 4.92, 95%CI: 4.33 ~ 5.58), experience of negative life events during pregnancy (First trimester: OR = 1.47, 95%CI: 1.07 ~ 2.02; Second trimester: OR = 2.19, 95%CI: 1.48 ~ 3.16; Third trimester: OR = 2.12, 95%CI: 1.47 ~ 2.99), as well as drinking before (First trimester: OR = 1.96, 95%CI: 1.70 ~ 2.26; Second trimester: OR = 1.83, 95%CI: 1.49 ~ 2.23; Third trimester: OR = 1.87 95%CI: 1.56 ~ 2.24) and during pregnancy (First trimester: OR = 1.82, 95%CI: 1.66 ~ 2.01; Second trimester: OR = 1.60, 95%CI: 1.39 ~ 1.85; Third trimester: OR = 1.44, 95%CI: 1.26 ~ 1.64).

Factors associated with a reduced risk of depressive symptoms in all the three trimesters were daily physical activity (First trimester: OR = 0.48, 95%CI: 0.42 ~ 0.55; Second trimester: OR = 0.41, 95%CI: 0.31 ~ 0.52; Third trimester: OR = 0.68 95%CI: 0.56 ~ 0.83), and having one child (First trimester: OR = 0.73, 95%CI: 0.65 ~ 0.81; Second trimester: OR = 0.82, 95%CI: 0.69 ~ 0.97; Third trimester: OR = 0.80 95%CI: 0.69 ~ 0.94), two children (First trimester: OR = 0.58, 95%CI: 0.48 ~ 0.70; Second trimester: OR = 0.61, 95%CI: 0.45 ~ 0.81; Third trimester: OR = 0.56, 95%CI: 0.43 ~ 0.73), or three or more children (First trimester: OR = 0.19, 95%CI: 0.11 ~ 0.32; Second trimester: OR = 0.46, 95%CI: 0.21 ~ 0.89; Third trimester: OR = 0.48, 95%CI: 0.25 ~ 0.84) prior to the current pregnancy.

Several factors exhibited significant influence on depressive symptoms during specific trimester combinations. For instance, in the first and second trimester, higher education levels such as a bachelor’s degree (First trimester: OR = 1.60, 95%CI: 1.43 ~ 1.79; Second trimester: OR = 1.38, 95%CI: 1.16 ~ 1.64) and a master’s degree or above (First trimester: OR = 2.07, 95%CI: 1.70 ~ 2.51; Second trimester: OR = 1.71, 95%CI: 1.26 ~ 2.30), unplanned pregnancy (First trimester: OR = 1.09, 95%CI: 1.00 ~ 1.17; Second trimester: OR = 1.21, 95%CI: 1.08 ~ 1.36), smoking during pregnancy (First trimester: OR = 1.43, 95%CI: 1.04 ~ 1.93; Second trimester: OR = 1.69, 95%CI: 1.12 ~ 2.49), as well as husband drinking before (First trimester: OR = 1.49, 95%CI: 1.36 ~ 1.62; Second trimester: OR = 1.57, 95%CI: 1.38 ~ 1.80) and during pregnancy (First trimester: OR = 1.47, 95%CI: 1.26 ~ 1.71; Second trimester: OR = 1.61, 95%CI: 1.29 ~ 2.02) were predictors of higher PHQ-9 scores; while engaging in physical activity occasionally (First trimester: OR = 0.59, 95%CI: 0.55 ~ 0.64; Second trimester: OR = 0.84, 95%CI: 0.75 ~ 0.95), or weekly (First trimester: OR = 0.38, 95%CI: 0.33 ~ 0.42; Second trimester: OR = 0.60, 95%CI: 0.50 ~ 0.73) were associated with lower risk. In the first and third trimester, living with the women’s own parents was linked to an increased risk of depressive symptoms (First trimester: OR = 1.31, 95%CI: 1.16 ~ 1.53; Third trimester: OR = 1.34, 95%CI: 1.08 ~ 1.64).

Certain factors were influential only in one specific trimester. For example, in the first trimester, younger age (16 ~ 25) (OR = 0.80, 95%CI: 0.72 ~ 0.88) and an annual family income above CNY 240,000 (OR = 0.52 95%CI: 0.35 ~ 0.78) was associated with a reduced risk of depressive symptoms; while living alone was associated with an increased risk (OR = 1.34, 95%CI: 1.05 ~ 1.69). In the second trimester, assisted conception (OR = 1.38, 95%CI: 1.09 ~ 1.73]) and smoking before pregnancy (OR = 1.78, 95%CI: 1.27 ~ 2.46) were linked to an increased risk. In the third trimester, husband smoking before ( OR = 1.34, 95%CI: 1.19 ~ 1.51) and during pregnancy (OR = 1.39, 95%CI: 1.14 ~ 2.70) were linked to an increased risk.

## Discussion

This study of 110,584 pregnant women enrolled in a mobile app-based screening programme in Shenzhen presents the largest population-based investigation of antenatal depression in Mainland China to date. It revealed an overall prevalence of antenatal depressive symptoms of 18.0%, with the risk associated with a range of sociodemographic, psychological, obstetric, and lifestyle factors. Some of these findings corroborate those of previous studies, whereas others present novel predictors of antenatal depression, enriching the current understanding of this condition.

### Prevalence of antenatal depressive symptoms

The prevalence of antenatal depressive symptoms identified in this study (18.0%) falls within the range of prevalence (15.8% to 24.2%) reported in existing literature, as indicated by Nisar et al.’ systematic review [17]. The substantial sample size of 110,584 pregnant women, representing 82.3% of all women who gave birth during the study period in Shenzhen, provided a robust dataset, likely reflecting a more accurate estimation of the depression rate. Moreover, this prevalence is lower than the rates reported from other parts of China during the COVID-19 pandemic [35, 36], appearing not to be affected by the COVID-19 pandemic. Only 4.2% of the women exhibited PHQ-9 scores of 10 or higher at least once during the three trimesters, indicating a low prevalence of high-level antenatal depressive symptoms. This finding is similar to a recently published study in Hunan Province, central China, which found that only 62 (3.3%) out of 1862 postpartum women who screened positive for depression were diagnosed with a current depressive condition using a structured diagnostic interview [37].

Furthermore, this study found that only a very small proportion of women (0.4%) were screened positive across all trimesters, which is significantly lower than the rates reported in published studies. For example, a study of 996 Chinese pregnant women identified 107 (10.7%) with persistent depressive symptoms in their second and third trimesters [38]. In a cohort study of 1813 pregnant women in the United States, only 4% reported persistent depressive symptoms throughout pregnancy [39]. Such variation might be due to differences in study design, population characteristics, and the impact of interventions received. It also highlights the complex and multifaceted nature of antenatal depression, with variations in the onset, duration, and configuration of symptoms among different women [40]. Some studies have explored the trajectory of depressive symptoms among Chinese pregnant women, finding that different trajectory groups may have varying risk factors and impacts on maternal and infant health [40, 41].

The findings suggest that depressive symptoms were most prevalent in the first trimester (14.3%) and decreased in the second (7.7%) and third trimesters (7.7%). This pattern is consistent with a recent study in Chengdu, an economically developed city in Southwest China, which also highlighted early pregnancy as a particularly vulnerable period [41]. The higher depression rates during this time may be attributed to multiple factors, including the women’s physical discomfort and severe pregnancy-related sickness like nausea and hyperemesis gravidarum, as well as concerns over spontaneous abortions and fetal abnormalities [41]. As pregnancies progress into the second and third trimesters and women receive more comprehensive antenatal care and information about fetal health, their ability to adapt to the pregnancy improves, leading to a diminished risk of depression. Additionally, the decreased prevalence in Shenzhen may be partly due to the fact that women who screened positive in the first trimester might have received some extent of follow-up or psychological interventions, potentially easing their depressive symptoms in the following trimesters.

### Factors associated with antenatal depressive symptoms

Statistical analysis in this study identified anxiety symptoms in early pregnancy as the most significant predictor of antenatal depressive symptoms, showing a markedly increased risk of 78.99 times. Although this rate decreased to 6.09 and 4.39 times in the second and third trimesters, it remained higher compared to other factors. This finding aligns with both national and international evidence. A meta-analysis of 95 Chinese studies indicated that maternal anxiety symptoms could amplify the risk of antenatal depression by 2.604 to 19.987 times [17]. Similarly, a global systematic review pinpointed anxiety symptoms as a leading predictor of antenatal depressive symptoms [42]. Several factors were also identified as associating with an increased risk throughout pregnancy, including lower marital satisfaction, living with parents-in-law, experiencing negative life events, and substance use such as drinking and smoking before and during pregnancy. These observations are supported by a broad spectrum of literature. Various Chinese studies have shown that lower marital satisfaction and living with parents-in-law are associated with increased risks of antenatal depression, quantified as 0.331 to 4.772 times [18, 19, 41, 43] and 0.319 to 2.55 times [19, 20, 38, 41], respectively. Conflicts with in-laws can serve as chronic stressors, potentially worsening women’s mental health and straining marital relationships, thus reducing spousal support—another recognised risk factor for perinatal depression [38, 44]. Negative life events, acknowledged in both national and international research, contribute psychological and economic stress that significantly impacts the risk of antenatal depression [18, 41, 42, 45]. A meta-analysis of 173 global studies found that smoking before and during pregnancy is linked to 1.97 and 2.04 times of antenatal depression risk [24]. A recent study in Northwest Ethiopia revealed that pregnant women with a history of alcohol use were 2.41 times more likely to exhibit depressive symptoms compared to those without [23].

In addition, this study marks the first extensive assessment in China of the relationship between husbands’ substance use and antenatal depression. It found that pregnant women whose husbands engaged in drinking or smoking were more likely to experience depressive symptoms during pregnancy. This finding aligns with existing research. A study conducted in Chengdu found that poor marital relationships, often exacerbated by husbands' smoking and drinking, were significant predictors of antenatal depression [38].

Remarkably, this study found that pregnant women with a bachelor's degree or higher faced an increased risk of antenatal depressive symptoms in the first and second trimesters. This unexpected correlation, contrary to previous research findings [19, 20], might reflect the pressures and stress associated with balancing a demanding career and the challenges of pregnancy, as well as confounding factors not directly observed in this study [46]. Shenzhen is among the most developed cities in China, with a large proportion of its population being younger, more educated, and career-oriented women. Highly educated women may occupy higher positions, leading to increased stress and, consequently, higher rates of depressive symptoms. This finding is consistent with a published study on the predictors of postpartum depression in the city [5]. Self-employed or business women were more likely to experience depressive symptoms in the second and third trimesters, possibly due to the unique stressors of managing a business, such as financial instability and lack of maternity leave. Unlike prior research findings [18, 47, 48], lower family income levels, unplanned pregnancy, and assisted conception were not found to be associated with higher risk of antenatal depressive symptoms in this study.

This study discovered multiparity—having given birth to one or more children before—is inversely related to the likelihood of experiencing depressive symptoms during pregnancy. This correlation suggests that with each subsequent birth, women may develop stronger coping mechanisms to manage childbearing and parenting stress, thereby reducing their risk of depression. Additionally, engaging in physical activity, even occasionally, correlated with reduced risk of depressive symptoms, with more frequent exercise further lowering the risk. This is supported by a systematic review by Kołomańska et al., indicating that physical activity, at least once a week, significantly reduces depressive symptoms in pregnant women and is crucial for the prevention of antenatal depression [49].

### Strengths and limitations

The study's use of health information system data with a large sample size enhances the statistical power and representativeness of findings on antenatal depression in Shenzhen, offering insights that may be more reflective of broader realities. However, the large sample also led to almost every variable showing statistical significance in logistic regression, some of which might lack practical relevance [50]. Another limitation of this study is that it only included women who gave birth in Shenzhen and underwent depression screening at least once during pregnancy. The exclusion of women who did not complete the screening or gave birth outside of Shenzhen may have introduced selection bias, potentially underestimating the true prevalence of antenatal depressive symptoms. Additionally, the cross-sectional design precludes establishing causal and temporal relationships. While the research provides a comprehensive analysis of antenatal depressive symptoms and their predictors across trimesters, it overlooks crucial factors such as social support [2, 23], experience of intimate violence [42, 47], and sleep quality [2, 43], all of which are significant predictors of antenatal depression. Moreover, comparing the identified predictors of antenatal depressive symptoms with those from other studies is challenging due to differences in definitions, factor compositions, and statistical methodologies [2, 17].

### Implications

This study offers practical implications for the planning of universal screening and intervention strategies within the new policy context of China. First, leveraging mobile technology could streamline the integration of depression screening into routine antenatal care, ensuring extensive reach. Second, focused interventions are recommended for pregnant women with existing mental health conditions, particularly anxiety symptoms in early pregnancy, or those with higher education levels, less birth experience, lower marital satisfaction, experience of negative life events during pregnancy, or residing with parents-in-law. Thirdly, implementing depression-related prenatal education is crucial, promoting healthy lifestyles among pregnant women, such as regular physical activity, and advocating for supportive behaviours from their husbands, including quitting smoking and drinking. Fourthly, given a 18.0% overall prevalence, the fact that only 0.4% of pregnant women exhibited persistent depressive symptoms across all trimesters underscores the importance of dynamic monitoring of depressive symptoms during pregnancy. Finally, screening is just the initial step. Ensuring that women identified at risk receive comprehensive follow-up and are directed towards appropriate psychological or psychiatric services is paramount. The use of health information systems for efficient management and monitoring of this process is vital, as is conducting further research to examine referral uptake among Chinese women with high level of antenatal depressive symptoms and identifying barriers to healthcare access.

## Conclusions

This study, involving 110,584 pregnant women in Shenzhen screened via a mobile app, discovered a 18.0% overall prevalence of antenatal depressive symptoms, with trimester-specific prevalences being the highest in the first trimester. Anxiety symptoms in early pregnancy was identified as the most significant predictor. The results highlighted the importance of targeted interventions for women with existing mental health conditions, higher education levels, lower marital satisfaction, living with parents-in-law, experiencing negative life events, and having substance use issues either personally or by their husbands. The study underscores the necessity of dynamically monitoring depressive symptoms for pregnant women and ensuring at-risk women receive comprehensive follow-up and appropriate psychological or psychiatric care.

## Data Availability

Data supporting the findings of this study is not available due to the data policy of Shenzhen’s perinatal depression screening and intervention programme, and therefore will not be deposited in a public repository. Access to the data may be requested from the corresponding author on reasonable grounds, subject to approval by the Medical Ethics Committee of Shenzhen Maternity and Child Healthcare Hospital.

## Abbreviations

AD: Antenatal depression
PHQ-9: 9-Item Patient Health Questionnaire
ACOG: American College of Obstetricians and Gynecologists
MCHMIS: Maternal and Child Health Management Information System
CNY: Chinese yuan
GAD-7: 7-Item General Anxiety Disorder scale
OR: Oddis ratios
CI: Confidence interval
